# Cross‐Cultural Variation in the Noun Bias in Early Vocabulary Development: A Systematic Review

**DOI:** 10.1111/cogs.70241

**Published:** 2026-07-09

**Authors:** Yiqun Zhang, Subin Kim, Marisa Casillas

**Affiliations:** ^1^ Department of Psychology University of Pennsylvania; ^2^ Department of Comparative Human Development University of Chicago

**Keywords:** Noun bias, Word learning, Vocabulary, Systematic review, Cross‐linguistic comparison, Vocabulary checklist, Typology

## Abstract

Children's early vocabularies have been observed to be predominated by nouns, particularly concrete and animate nouns. This phenomenon, known as the “noun bias,” is argued to reflect a universal conceptual advantage for concrete objects in early word learning, because such referents are perceived as bounded, individualized concepts that are ready to be mapped onto linguistic labels. The noun bias has been observed across a wide range of languages using multiple methodological approaches, establishing it as a robust feature of early lexical development. However, the magnitude and consistency of the noun bias have also been observed to vary across linguistic and cultural contexts, motivating further investigation of the factors that shape this phenomenon. The present study systematically reviews and quantitatively analyzes evidence from 68 published and unpublished papers spanning 28 languages over five decades (1973–2025) to examine how the noun bias is shaped by linguistic and other properties. The review suggests general evidence supporting a noun bias. However, research methodology and noun morphology emerge as significant predictors of variation in the observed noun bias magnitude. In addition, the presence of specific linguistic attributes—noun plural marking and verb past tense marking—explains variation in the observed noun bias outcomes from the 68 papers reviewed. This comprehensive view of the literature sheds light on limitations and inconsistencies in the literature that should be remedied in future work to further advance theoretical accounts of the noun bias.

## 1 Introduction

Young children tend to learn words for concrete objects before words for abstract relations (e.g., Au, Dapretto, & Song, 1994; Bassano, Maillochon, & Eme, 1998; Gentner, 1982; 2006; MacNamara, 1972; Nelson, 1973; Setoh, Cheng, Bornstein, & Esposito, 2021; see Waxman et al., 2013, for a review), a pattern widely known as the “noun bias.” In fact, children's early mastery of these words is argued to form a basis for their later acquisition of more abstract and relational words, including verbs, modifiers, and also words whose function is primarily syntactic (e.g., Gentner's (1982) “hooks” and Gleitman's (1990) syntactic bootstrapping mechanism). While theories differ in their proposed origins of the bias, and what dependencies they introduce into the process of building a lexicon, cumulative evidence over the past 50 years underscores the consistency of this developmental phenomenon. Indeed, recent work using a semi‐standardized parental report measure of expressive vocabulary across typologically diverse languages shows a relative overrepresentation of nouns in all 22 languages included (Braginsky, Yurovsky, Marchman, & Frank, 2019; Frank, Braginsky, Yurovsky, & Marchman, 2021).

This observed early bias for nouns is taken as strong evidence for a conceptual underpinning of early word learning—that individuable, concrete referents are naturally salient *and* well‐suited for the categorical labels used by linguistic systems (Gentner, 1982; 2006; Macnamara, 1972; alternatively, see Gillette, Gleitman, Gleitman, & Lederer, 1999). In her foundational work on this topic, Gentner (1982; 2006) formulates the natural partitions/relational relativity (NP/RR) hypothesis, to explain the overrepresentation of nouns in children's early productive vocabularies, along with observed variation across languages in how action versus object concepts are expressed. Under the NP/RR account, children are subject to the noun bias due to the universal conceptual availability of concrete object referents, allowing small variations in observed noun bias size due to structural differences across languages. Several other ideas have been put forth to explain the predominance of object words in children's early vocabularies, including Gillette et al.’s (1999) proposal that, while concrete object nouns typically only require word‐to‐world mappings, verbs require sentence‐to‐world mappings, and thus depend on the prior acquisition of some basic syntactic knowledge (not conceptual maturation; see also Gleitman, 1990). Another argument is that children's noun use simply echoes patterns from their input (e.g., for sociocultural or linguistic reasons, such as an emphasis on object labeling in U.S. child‐directed speech; de León, 1999; Gentner, 1982; Tardif, 1996). However, prior work suggests an asymmetry between the noun predominance of adult language input and children's own language production (e.g., Bates et al., 1994; Choi & Gopnik, 1995; Gopnik & Choi, 1995).

Each account for the noun bias relies on some core mechanism to explain its appearance; however, the magnitude of the noun bias is well known to vary substantially across cultural, linguistic, and interactional contexts (e.g., Bornstein et al., 2004; Braginsky et al., 2019; Brown, 1998; de León, 1999; Frank et al., 2021; Gentner, 1982; M. Kim, McGregor, & Thompson, 2000; Setoh et al., 2021; Tardif, 1996; Tardif, Gelman, & Xu, 1999). For instance, one crosslinguistic study of early word production reported a much stronger noun bias in U.S. English than Korean, with individual U.S. children's noun:verb ratios ranging from 5:1 to 33:1, compared to Korean children's ratios ranging from 1.1:1 to 2.9:1 (M. Kim et al., 2000). Some prior research has even found a “verb bias,” a pattern perhaps especially likely when learning a “verb‐friendly” language (Gentner, 1982). Verb‐friendly languages have structural features that can emphasize verbs in children's linguistic input, for example, relatively greater verb frequencies (e.g., via noun ellipsis in pro‐drop languages), more salient verb utterance positions (e.g., verb‐initial and verb‐final utterances, see also Slobin, 1985), or approximately equal use of stem‐altering morphology across verbs and nouns (e.g., Brown, 1998; Tardif, Shatz, & Naigles, 1997). For example, Tardif's (1996) observational study of 22‐month‐old Mandarin‐speaking children found a slight verb bias, with 9 out of 10 children producing more main verbs than common nouns. Gopnik and Choi (1995) and Choi and Gopnik (1995) found no clear evidence for a noun bias in Korean‐acquiring children; but they *did* see evidence for children's early verb productivity and verb‐friendly input. Observational studies of Tseltal and Tsotsil, both Mayan languages with verb‐friendly features and action‐oriented caregiver input, found no overrepresentation of nouns, finding instead, slight evidence for a verb bias (Brown, 1998; Casillas, Foushee, Girón, Polian, & Brown, 2024; de León, 1999; see also Stoll et al., 2012 on Chintang).

These claims have not stood unchallenged; counterevidence in one or more noun bias measurements has been presented for Mandarin, Korean, and Tseltal, showing some evidence for a noun bias in all three languages despite their “verb‐friendly” characteristics (e.g., Mandarin: Gentner, 1982; Tardif et al., 1999; Korean: Au et al., 1994; Pae, 1993; Tseltal: Brown, Gentner, & Braun, 2005 as cited in Gentner, 2006). In some cases, the apparently mixed evidence for the noun bias—within these and other languages—has been attributed to differences in methodological approach. Noun bias estimates tend to be higher for checklist‐based studies than observational ones (Pine, 1992; Pine, Lieven, & Rowland, 1996; Tardif et al., 1999).

This degree of observed variation calls for a systematic check on the factors that drive differences in early word learning, particularly linguistic structure, developmental environment, and interactional context, as important factors shaping young children's lexical development. These factors likely integrate with any basic conceptual biases to shape attention toward potential word referents. The present study quantitatively and systematically reviews previous evidence for variation in noun bias magnitude using both top‐down and bottom‐up analyses of prior findings to identify the linguistic and methodological attributes significantly associated with noun bias magnitude.

### 1.1 Linguistic attributes

The possibility of “verb‐friendly” languages is well acknowledged in the literature (e.g., Au et al., 1994; Gentner, 1982; 2006; Gopnik & Choi, 1990; M. Kim et al., 2000; Ogura, Dale, Yamashita, Murase, & Mahieu, 2006; Tardif, 1993; Tardif et al., 1997). As mentioned above, such languages are thought to have typological or input features that facilitate early verb learning, including: greater verb predominance (e.g., via noun ellipsis; Caselli et al., 1995; M. Kim et al., 2000; Tardif et al., 1997), verb position at the utterance edge or variable basic word order (BWO) (e.g., Caselli et al., 1995; Slobin, 1985), and more equal stem‐altering morphosyntax on verbs compared to nouns (e.g., Brown, 1998; Gentner, 1982; Tardif et al., 1997). We elaborate on each of these linguistic attributes and how they might be linked to a lesser noun bias below.

While one might generally expect nouns to be more frequent than verbs (e.g., because transitive events involve two or more objects and one action), the opposite has been reported for spontaneous speech (e.g., Sandhofer, Smith, & Luo, 2000); differences in noun and verb frequency between languages may be instead linked to the permitted extent of noun ellipsis in a given language. Note that “pro‐drop” is then best conceptualized as a gradient feature rather than a binary one by virtue of the noun ellipsis phenomenon. For example, Korean allows more noun ellipsis than English (Au et al., 1994) and thus verbs are relatively more prominent in Korean‐speaking children's linguistic environments than English‐speaking children's, as evidenced by higher verbal type and token frequency (average proportions for Korean verb‐noun types = 58.87% and tokens = 54.66% vs. English types = 44.04% and tokens = 49.16%; M. Kim et al., 2000). Noun ellipsis increases the relative frequency of verbs, and may also result in more frequent isolation of verb forms, both of which may support greater verb salience and earlier verb acquisition. However, evidence from cross‐linguistic novel word learning experiments suggests that noun ellipsis may actually hinder novel verb learning (Arunachalam & Waxman, 2011; Gleitman, Cassidy, Nappa, Papafragou, & Trueswell, 2005). The authors argue that children in fact *rely* on the semantic and syntactic properties of surrounding nouns for word learning (Gleitman, 1990), and so the frequent dropping of nouns in “verb‐friendly” languages may instead lead to a disadvantage in verb learning. Ultimately, however, languages differ in when and why nouns are elided (e.g., see Tardif et al. (1997)'s discussion on pro‐drop in Italian and Mandarin). It is thus difficult to know how frequently ellipsis occurs in children's linguistic input without a sizable corpus analysis.

Utterance position is another linguistic feature linked to word learning due to the “edge effect,” which suggests that words at syntactic boundaries are perceptually more salient and are hence more likely to be attended to by infants (Johnson & Seidl, 2008; Seidl & Johnson, 2006; Slobin, 1985). In English, a language with strong evidence for a noun bias, the BWO is subject‐verb‐object (SVO), with nouns canonically placed at the initial and final edges of utterances. Approximately half of the languages documented in the presently largest online repository of grammars (Grambank; Skirgård et al., 2023) have a verb‐medial word order in transitive clauses (1109/2433, 45.6%^1^). However, other languages have verb‐initial (e.g., 470/2448, 19.2%; Tseltal VOS; Polian, 2018) and verb‐final BWOs (e.g., 1014/2446, 41.5%; Korean SOV; Yeon & Brown, 2013), in which verbs have a greater edge advantage in word learning compared to verb‐medial languages like English and more free word‐order languages, such as Turkish (Öztürk, 2008), which may fall somewhere in between. Notably, languages differ in how rigidly they adhere to their default word order, and verbs may appear at the utterance edges because of other processes (e.g., see remarks on elision above; see also Levshina et al., 2023, for more on the theoretical implications of gradience in word order). Thus, reliable estimates for noun and verb utterance‐edge appearance should ideally be derived from substantial corpora of child language input (Braginsky et al., 2019; Caselli et al., 1995; Stoll et al., 2012).

A final linguistic factor investigated in past noun bias research is morphosyntactic complexity—with the idea that morphosyntax hinders early word learning (Gentner, 1982; Stoll et al., 2012; Tardif et al., 1997; Tyler, 2004). Since verbs tend to carry more morphology than nouns do, their acquisition is thought to require greater grammatical competence (Mithun, 2019; see also Gleitman, 1990 and Gillette et al., 1999). Languages vary in how much inflectional morphology is added to nouns, verbs, and other word types, allowing for the natural exploration of this idea. Stoll et al. (2012) suggest that the rich verbal morphology of Chintang—a highly polysynthetic language—combines with children's play talk to cause less frequent verb production in child talk, compared to adults. Chintang children's noun bias is negatively correlated with their grammatical competence. Following similar reasoning, others have argued that languages with minimal verbal inflection (e.g., isolating languages, like Mandarin) help neutralize the morphosyntactic simplicity advantage of nouns over verbs, leading to a lessened noun bias (Tardif et al., 1997; see also Brown, 1998, on Tseltal). On the other hand, scant morphology may also cause greater difficulty in distinguishing between the grammatical forms of nouns and verbs, hindering overall word learning (Imai et al., 2008).

Thus, precisely how morphosyntactic complexity relates to noun bias is yet underspecified. Even if we accept the basic assumption that more morphosyntactic inflection leads to hindered word learning, there are several ways in which a noun bias might arise: when languages (a) have very little morphosyntax on nouns, (b) have substantial morphosyntax on verbs, or (c) have substantially more morphosyntax on verbs compared to nouns. These predictions may differ for agglutinating versus fusional languages, and would differ still for languages with a great deal of suppletion. Comparing Chintang with Tseltal, Stoll et al. (2012) also suggest that more morphology is not always a hindrance; Tseltal's more clitic‐like morphosyntax may well *facilitate* stem segmentation, whereas Chintang's more affixal morphology hinders it. Indeed, one might hypothesize that (regular) inflectional morphology could overall *aid* word learning by facilitating stem segmentation (e.g., Y. J. Kim & Sundara, 2021; Marquis & Shi, 2012; Mintz, 2013; but see Frost, Dunn, Christiansen, Gómez, & Monaghan, 2020, on novel anchors). In sum, morphosyntactic complexity is a relevant linguistic attribute in understanding how the noun bias may arise, but the present theory does not yet point to which morphological characteristics might matter the most.

### 1.2 Methodological attributes

Methodology has long been argued to drive variation in observed noun biases (Pine, 1992; Pine et al., 1996; Tardif, 1996). Three primary methods have been used: (1) observational analysis of children's (and parents’) spontaneous language production; (2) retrospective parent‐reported accounts of children's vocabulary (e.g., MacArthur−Bates Communicative Development Inventories, “CDI”; E. Bates et al., 1994; Marchman, Dale, & Fenson, 2023); and (3) controlled experiments that shed light on children's word‐learning mechanisms and existing knowledge, typically via novel word learning.

The analysis of spontaneous speech transcripts—from recordings made at home (Tardif, 1996) or in laboratory settings (Setoh et al., 2021; Snedeker, Li, & Yuan, 2003; Tardif et al., 1999)—is easily applied across culturally and linguistically diverse communities. Natural observation gives simultaneous insight into children's vocabulary, their linguistic input, and the influence of interactional context on word use (e.g., Goldenberg, Repetti, & Sandhofer, 2022; Hoff, 2010; Tardif et al., 1997). However, this method typically only captures a small slice of a child's total expressive vocabulary and may undershoot noun bias estimates detected via other methods (Aspland & Gardner, 2003; Caselli et al., 1995; Gentner, 2006). Following prior work, one could aim to systematically sample multiple activity contexts across distinct language communities (e.g., English and Mandarin; Tardif et al., 1999). But beyond a handful of major languages, we do not have access to transcribed, systematically context‐variable interaction data. Indeed, creating such a dataset for the dozens of languages in our present sample would have immense value for the field. Because there is no set of activity contexts that we can expect to have equal relevance, familiarity, and composition across developmental age and diverse cultural contexts, we would have to make such comparisons on the basis of very dense and comprehensive datasets documenting children's linguistic input and early speech production (Casillas, 2023). We are, however, practically limited from achieving this goal at present. Manual transcription costs a great deal of time and money (Casillas, 2023; Montag et al., 2025), and automated tools are not yet up to the task of transcribing naturalistic at‐home speech (typically producing a ∼40–50% word error rate on representative naturalistic data in U.S. English and bilingual U.S. English/Spanish households; M. Lavechin, personal communication; but see Kocharov & Räsänen, 2025, for a preliminary workaround). Thus, transcription data are still practically limited to very small samples of children's total language experience.

Vocabulary checklists, like the CDI, provide a broader view of children's early receptive and expressive vocabulary, based on a caregiver's cumulative experience with their child. Checklists are associated with clearer evidence of noun bias (E. Bates et al., 1988; Pine, 1992; Pine et al., 1996) but may also be logistically less well suited for working with under‐represented or hard‐to‐reach communities (see Kachergis, Tan, Marchman, Dale, & Frank, 2026, for an alternative to full‐checklist adaptation). Suitability might be reduced in these cases because researchers are often working with smaller sample sizes of participants, there may not be a standard orthography of the language, and the researcher may be minimally motivated to collect data that has highly restricted potential for reuse compared to transcript data (which can be analyzed again and again for a wide variety of phenomena). In short, researchers working with under‐represented communities may require significant time, knowledge of the language, and financial/personnel resources before checklists become a practical approach for measuring expressive vocabulary. Moreover, because checklists are retrospective reports rather than direct measures, they are subject to parents’ own biases (Nelson, Hampson, & Shaw, 1993), which may be difficult to control for when conducting a comparative analysis of different social, linguistic, or cultural groups.

The third common method, controlled experiments, typically uses images or videos to elicit labels (Colombo, Navarrete, & Arfé, 2017; Klassert, Gagarina, & Kauschke, 2014; Masterson, Druks, & Gallienne, 2008; Nilipour, Shirazi, Afshordi, & Kauschke, 2013), points (Arunachalam, Leddon, Song, Lee, & Waxman, 2013; Leddon et al., 2011), or looking time (Katerelos, Poulin‐Dubois, & Oshima‐Takane, 2011; Oshima‐Takane, Ariyama, Kobayashi, Katerelos, & Poulin‐Dubois, 2011; Waxman, Lidz, Braun, & Lavin, 2009) for depicted objects and actions. Experiments are deployed to test specific hypotheses about real‐time mechanisms for mapping form to meaning—this is their empirical strength. By design, such tasks do *not* capture the natural flow of everyday language, which may be a major drawback for theories of learning that rely on highly contextual and cumulative learning mechanisms. However, experiments are key for shedding light on mechanisms linked to moment‐to‐moment learning, especially when the hypothesized learning mechanism implicates controllable linguistic and interactional cues (e.g., morphosyntax). Such findings have been mixed. For example, some experimental studies with children acquiring English, Japanese, and Mandarin (Imai et al., 2008; Imai, Haryu, & Okada, 2005) have shown cross‐linguistic evidence of difficulties in mapping novel verbs to actions, compared to successful mapping of novel nouns to objects. Other experimental work finds that Mandarin‐learning children have a relative advantage over English‐learning for mapping and discriminating novel actions (Chan et al., 2011; Chen et al., 2015). Work by Arunachalam and colleagues (Arunachalam & Waxman, 2011; Arunachalam et al., 2013; Leddon et al., 2011) also suggests that the linguistic environment most conducive to novel verb learning varies as a function of the child's native language. In their studies, Korean‐learning children more successfully mapped novel verbs to actions when noun phrases were dropped, while English‐learning children displayed the opposite pattern. Such cross‐linguistic, experimental evidence lays critical groundwork for understanding the language‐specific factors that impact real‐time identification and mapping of referents and their labels.

### 1.3 The current study

As summarized above, linguistic (e.g., ellipsis, BWO, and morphological complexity) and methodological attributes (e.g., observation, checklist, and experiment) influence the observed magnitude of children's noun bias. However, it is yet unclear how these attributes interact and measure up against each other. The present study synthesizes five decades of empirical noun bias research, using two analyses: (1) mixed‐effects linear regression, to test proposed predictors of the noun bias (e.g., BWO; “top‐down” analysis) and (2) a random forest analysis, to evaluate the relative importance of individual attributes (e.g., specific inflection types; “bottom‐up” analysis). By accounting for documented crosslinguistic and methodological variation, this work aims to shed light on the variable realization of noun bias across diverse contexts, and to identify high‐priority areas for future research.

After careful consideration, we decided to exclude experimental studies from this systematic review. Prior work has considered a wide variety of theoretical issues surrounding the noun bias, including conceptual development, syntactic development, and early verb learning. Two major empirical projects resonate throughout this literature. The first is to *document* the phenomenon in a wide variety of populations and contexts (“documentation”). The second is to *explain* how and when it arises (“process inference”). Quite a bit of the research mentioned above attempts to accomplish both of these goals, for example, by looking within bilingual individuals (Conboy & Thal, 2006; Levey & Cruz, 2003), comparing across multiple monolingual populations (Bornstein et al., 2004; Gentner, 1982; Tardif et al., 1997), and sampling across methods and activity contexts (Salerni, Assanelli, D'Odorico, & Rossi, 2007; Tardif et al., 1999; Taverna & Waxman, 2020). The joint goals of documentation and process inference can be fruitfully achieved in analyses of both spontaneous speech and vocabulary checklist data (e.g., Gentner's foundational (1982) comparative analysis). However, experiments are typically focused solely on the second goal: testing specific hypotheses about the mechanisms that drive noun‐biased learning. One further difference we noted when reviewing the literature is that observational and checklist studies produce differently formatted results than experimental studies on the noun bias. That is, observational and checklist studies yield estimates of inventory size across different syntactic classes (e.g., unique noun and verb types observed), while experimental studies report effect sizes (e.g., the effect of noun ellipsis on successful verb‐action mapping in novel word learning). We cannot conceptually or numerically integrate across these different measure types in a single quantitative analysis. Finally, we noted that observational and checklist studies covered a typologically and culturally wider range of language communities compared to experimental studies (to date). A priority of our present work was to include a greater diversity of languages when possible. Given the above considerations, we focused this systematic review on nonexperimental studies. A future meta‐analysis of experimental noun bias findings would nicely complement the present work.

## 2 Method

Study materials, analysis scripts, and a reproducible version of this manuscript can be found on OSF (https://osf.io/rkges/).

### 2.1 Literature search

**Fig. 1:**
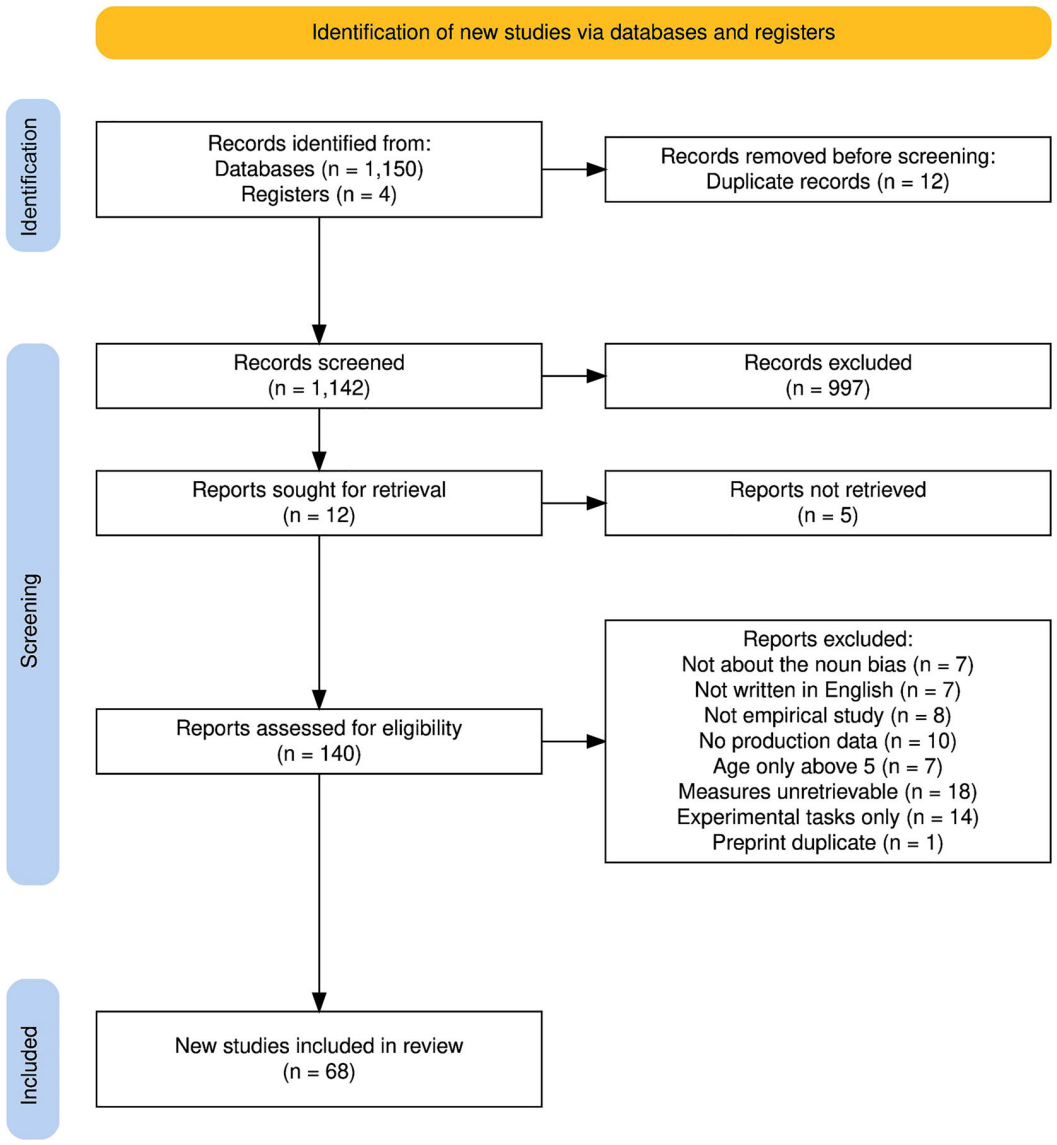
Flow chart of the study identification and selection process, following Preferred Reporting Items for Systematic Reviews and Meta‐Analyses (PRISMA) guidelines.

In November 2023, relevant published and unpublished studies were identified on Google Scholar by (a) using Publish or Perish software (Harzing, 2007) and (b) searching citations of Gentner (1982; 2006). Literature search was guided by the Preferred Reporting Items for Systematic Reviews and Meta‐Analyses (PRISMA) checklist (Fig. 1; Haddaway, Page, Pritchard, & McGuinness, 2022; PRISMA‐P Group et al., 2015). The following search terms were applied: “noun bias” OR “nominal bias” OR “noun advantage” OR “natural partition” OR “relational relativity” AND “child/infant language.”^2^ The search focused on studies written in English, since 1970, with participants under the age of 5 years.^3^

### 2.2 Eligibility criteria

Studies were deemed eligible for inclusion if they reported original research on the expressive vocabulary of typically developing children. To avoid publication bias, unpublished data from dissertations, theses, and conferences that fitted all inclusion criteria were also included. If a study contained data from children both under *and* above the age of 5 years, the study was included, with *only* data under the age of 5 years coded and analyzed. Experimenter‐controlled laboratory studies were excluded due to practical challenges in standardizing outcome measures. Papers (*N* = 1154) were screened by the primary coder (YZ) based on titles, abstracts, and full texts (Fig. 1), with 35% randomly selected for independent screening by a second coder (SK). Initial inter‐coder reliability was 95.5%, and discrepancies were resolved through discussion.

### 2.3 Data extraction and coding

**Fig. 2:**
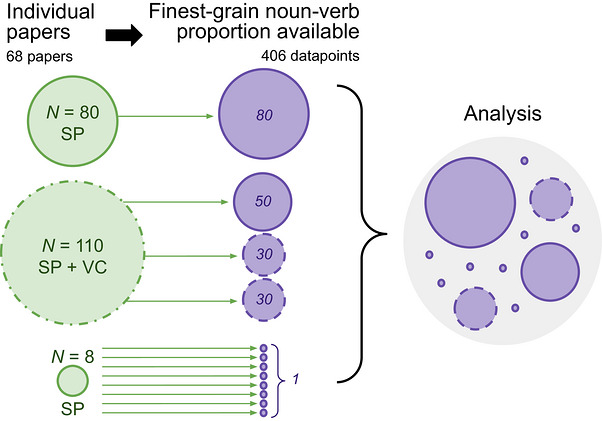
Noun and verb production datapoints were extracted at the finest granularity possible for each of the screened papers (406 datapoints from 68 papers). In this example, three hypothetical studies are shown: TOP = an 80‐participant spontaneous production (“SP”) study for which one grand average is reported; MIDDLE = a 110‐participant study with an SP component (one grand average reported) and a vocabulary checklist (“VC”) component (one average reported each for two age groups); BOTTOM = an 8‐participant two‐timepoint longitudinal SP study with outcomes reported for each participant at each timepoint. Finest granularity was defined on the basis of age (broken across longitudinal datapoints wherever possible), method, and language spoken.

The screened studies were coded for noun bias size, participant age, language, number of participants represented, method, and target syntactic categories at the finest granularity possible (Fig. 2). When studies reported only aggregated statistics for a group (e.g., a single productive vocabulary measure for an age group with a sample size of 10), we treated that aggregated vocabulary value as one datapoint and recorded the corresponding group‐average age and total group sample size (e.g., *N* = 10). However, we were able to extract disaggregated statistics from participant groups in quite a few papers, thanks to their authors’ detailed reporting of results. For example, some studies provided individual‐level data (e.g., productive vocabulary measures reported separately for each of the children in the study); we could then code each individual child's language measure and age as a separate datapoint with a sample size of one. For multilingual, cross‐sectional, and longitudinal studies, we applied this same principle by coding data for each language and age group sample at the finest level of granularity available. While the extracted sample sizes across the resulting 406 data points were highly variable (sample size *SD* = 69.37, range = 1–659), the majority of extracted sample sizes were small, with a median sample size of one (i.e., individual‐level data).

Our noun bias measure was recorded as noun‐verb proportion (# Types_N_ / (# Types_N_ + # Types_V_)). For example, if a given child was found to produce 45 unique nouns and 15 unique verbs, then their noun‐verb proportion would be 0.75 (i.e., 45/(45+15)). Any proportion over 0.5 indicates a noun bias. Proportional bias is commonly used in previous work (Cheung, 2005; Tardif et al., 1999) and helps to make maximal use of the data reported in the screened studies. Other noun bias measures, for example, relative representation (Frank et al., 2021), require information that is only sporadically available across studies (e.g., an estimate of total vocabulary size). Studies were variable in what information was reported, with some only giving the proportions of nouns, verbs, and other word types in relation to total vocabulary, while others gave raw counts of word types across studied classes (e.g., nouns and verbs). Some studies also differentiated proper and common nouns, reporting them separately (e.g., “apple” = common noun; “Susan” = proper noun), while others only included common nouns (e.g., “apple” = noun; “Susan” = NA) and others grouped them together (e.g., “apple” and “Susan” = both nouns). In the screened studies, common nouns and main verbs were the most commonly used definitions of “nouns” and “verbs”; the present study uses those same definitions.

**Fig. 3:**
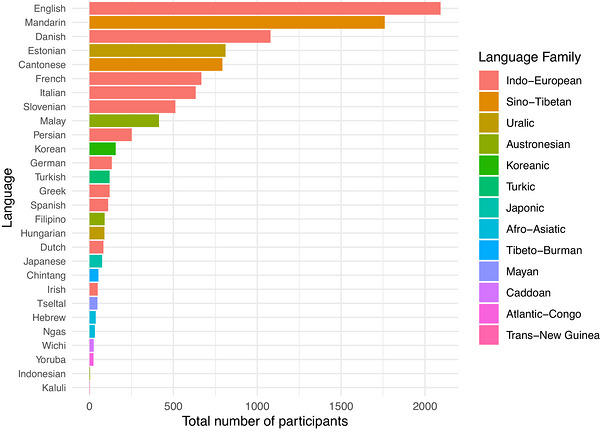
Distribution of 10,262 participants from 68 papers by language, sorted from the most to least well‐represented of the 28 languages in the literature, by total number of participants represented.

Morphological data for each language were extracted using Grambank, an electronic database of linguistic features (https://grambank.clld.org/; Skirgård et al., 2023). As of early 2026, Grambank includes 195 morphosyntactic features for 2467 languages across most basic grammatical domains (negation, tense, aspect, word order, argument marking, case, gender, number, etc.; Lesage, Haynie, Skirgård, Weber, & Witzlack‐Makarevich, 2022), including 25 morphosyntactic features relevant to the current study (i.e., noun and verb morphology; Table S1). Among these, 12 features were selected as “basic,” either because they relate to nominal number marking and diminutive/augmentative marking (six noun morphology features), or to tense, aspect, and mood (TAM) expression (six verb morphology features). We limited our use of Grambank features to these 12 basic features in the primary regression analysis because they indicate common inflection types for which we had metadata for nearly all of the languages.^4^ However, the 13 nonbasic morphosyntactic features *are* included together with the basic ones in the secondary, random forest analysis, because its nonparametric, bottom‐up approach is better suited to uneven data. Each language in the dataset was coded with a “1” indicating “yes” and a “0” indicating “no” for each of these 12 linguistic features, using “N/A” when the value was not available in Grambank. The “pro‐drop” variable was omitted in both analyses in accordance with our conceptualization of noun ellipsis as a largely gradient (i.e., nonbinary) phenomenon. Post‐hoc analyses confirmed that the exclusion of this variable did not affect our main results (Table S3). Nouns and verbs in each language were then given a proportional morphology score (e.g., for English: nouns [3 of 6 features; score = 0.5] and verbs [1 of 6 features; score = 0.17]). Unfortunately, actual rates of ellipsis and noun/verb frequency or utterance position in children's linguistic input could not be measured from each language—doing so would require data not currently available for all the languages found in the screened studies (e.g., via transcripts available on CHILDES; MacWhinney, 2000; Fig. 3).

Study method was coded as spontaneous production (observational), checklist‐based, or other. The present work focused on the first two approaches—observation and checklists—which comprise the majority of prior work (>90% of studies; see below).

A traditional meta‐analysis of the noun bias was not possible, due to most of the studies using methods that differed in design, measurement, and statistical approaches; there is typically no measure of effect size. Instead, the present work describes a quantitative systematic review of previously reported noun bias sizes. This approach gives a snapshot of the cumulative evidence regarding which linguistic and methodological attributes most strongly relate to observed noun biases.

### 2.4 Analytic approaches

To address the complexity of factors influencing the noun bias, this paper adopts two complementary analytic approaches: (1) a hypothesis‐driven (i.e., top‐down) regression analysis based on prior claims regarding influences on the size of the observed noun bias and (2) a data‐driven (i.e., bottom‐up) random forest analysis exploring the relative importance of individual linguistic and methodological attributes. The latter analysis is especially important to consider when trying to better understand the effects of morphosyntactic complexity; while there is a long‐standing intuition that morphosyntax matters for the noun bias, the specific morphosyntactic features that produce the bias are underspecified. Both analyses allow us to jointly examine linguistic and methodological effects; we can examine nonshared variance between these factors while trying to avoid overfitted interpretations from uneven datasets across methods.

## 3 Results

### 3.1 Descriptive analysis

The literature search process yielded 68 papers (Fig. 1 and Table 1) from which 406 interpretable noun‐bias datapoints were extracted, representing 10,262 children. Most (82.1%) of the children come from just eight languages, primarily English and Mandarin (Fig. 3). Spontaneous production and vocabulary checklist methods dominated the dataset, accounting for 74 (94.9%) studies from 64 papers (10,213 participants; > 99%). The two methods were roughly equally represented (spontaneous production: *N* = 38, 51.4%; vocabulary checklist: *N* = 36, 48.6%), but checklist studies had much larger sample sizes (mean = 176.7 vs. 33.5; median = 36 vs. 14; mode = 16 vs. 3). Checklist studies were also slightly less linguistically diverse, spread over nine language families (56.3% Indo‐European) compared to the 10 language families (43.5% Indo‐European) in spontaneous production studies.

**Table 1:** All 68 papers included in the systematic review, organized by language family, language, and year of publication

Authors (year)	Country	Language family	Language	Method	*N*	# Datapts.	Avg. age (range)
Childers, Vaughan, and Burquest (2007)	Nigeria	Afro‐Asiatic	Ngas	VC	32	4	22.5 (14–31)
Arokoyo (2012)	Nigeria	Atlantic‐Congo	Yoruba	SP	24	24	19.5 (16–23)
Tan‐de Ramos (2023)	Mania	Austronesian	Filipino	Other	30	1	48 (48–48)
Taverna and Waxman (2020)	Argentina	Caddoan	Wichi	VC, SP	25	6	30.33 (15–44)
Wehberg et al. (2007)	Denmark	Indo‐European	Danish	VC	1079	6	NA
Nelson (1973)	US	Indo‐European	English	SP	18	1	19.75 (19.75–19.75)
Goldin‐Meadow, Seligman, and Gelman (1976)	US	Indo‐European	English	SP	3	1	20.1 (20.1–20.1)
Bloom, Tinker, and Margulis (1993)	US	Indo‐European	English	SP	28	2	16.54 (13.84–19.23)
Dapretto (1994)	US	Indo‐European	English	VC	57	5	19.1 (9.45–23.55)
Goldfield (2000)	England	Indo‐European	English	SP	44	1	14 (14–14)
Li and Fang (2011)	England	Indo‐European	English	SP	12	1	22 (22–22)
Quigley and Nixon (2024)	Ireland	Indo‐European	English	SP	168	2	24.11 (24.11–24.11)
Bassano et al. (1998)	France	Indo‐European	French	SP	24	2	25 (20–30)
Bassano (2000)	France	Indo‐European	French	SP	5	5	21.9 (15.5–28)
Bassano, Eme, and Champaud (2005)	France	Indo‐European	French	SP	60	3	25.21 (17.27–38.24)
Kern (2007)	France	Indo‐European	French	VC	548	1	NA
Kauschke and Hofmeister (2002)	Germany	Indo‐European	German	SP	128	4	21.25 (13–36)
Chadjipapa (2021)	Greece	Indo‐European	Greek	SP	120	1	54 (54–54)
O'Toole and Fletcher (2012)	Ireland	Indo‐European	Irish	VC	49	4	26.5 (17–36)
D'Odorico, Carubbi, Salerni, and Calvo (2001)	Italy	Indo‐European	Italian	VC	126	3	21.17 (19.75–22.8)
D'Odorico and Fasolo (2007)	Italy	Indo‐European	Italian	VC	48	2	21.65 (17–26.3)
Salerni et al. (2007)	Italy	Indo‐European	Italian	VC, SP	82	4	24.2 (23.1–25.3)
Longobardi, Rossi‐Arnaud, Spataro, Putnick, and Bornstein (2015)	Italy	Indo‐European	Italian	SP	52	2	17 (16–18)
Longobardi et al. (2016)	Italy	Indo‐European	Italian	SP	90	3	19.67 (15.5–23.6)
Ebtedaei, Paknazar, Ghorbani, and Salmani (2023)	Iran	Indo‐European	Persian	VC	252	9	16 (12–20)
Marjanovič‐Umek, Fekonja‐Peklaj, and Podlesek (2013)	Slovenia	Indo‐European	Slovenian	VC	512	3	20.5 (12.5–28.5)
Caselli et al. (1995)	Multiple	Indo‐European	Multiple	VC	854	2	12.16 (12–12.31)
Conboy and Thal (2006)	US	Indo‐European	Multiple	VC	128	2	20.13 (20.13–20.13)
Xuan and Dollaghan (2013)	US	Indo‐European	Multiple	VC	100	2	26.9 (26.9–26.9)
Yamashita (1995)	Japan and US	Japonic	Japanese	Other	16	2	19 (17–21)
Miyata et al. (2003)	Japan	Japonic	Japanese	SP	24	24	19 (14–24)
Ogura et al. (2006)	Japan	Japonic	Japanese	SP	31	3	20.13 (17.4–22.9)
Pae (1993)	Korea	Koreanic	Korean	VC	90	3	17.33 (13.5–21.5)
Choi (1998)	US	Koreanic	Korean	VC	10	2	21.66 (19.23–24.1)
Brown (1998)	Mexico	Mayan	Tseltal	SP	6	6	25.99 (21.1–29.26)
Casillas et al. (2024)	Mexico	Mayan	Tseltal	SP	41	1	21.56 (21.56–21.56)
Leung (1998)	Hong Kong	Sino‐Tibetan	Cantonese	SP	6	6	21.17 (19–24)
Poon (1999)	Hong Kong	Sino‐Tibetan	Cantonese	VC	31	3	23 (17–29)
Cheung (2005)	Hong Kong	Sino‐Tibetan	Cantonese	SP	10	1	20 (20–20)
Tse, Chan, and Li (2005)	Hong Kong	Sino‐Tibetan	Cantonese	SP	328	2	42 (36–48)
Tardif (1993)	US	Sino‐Tibetan	Mandarin	SP	20	2	23.75 (21.8–25.7)
Tardif (1996)	China	Sino‐Tibetan	Mandarin	SP	10	1	21.77 (21.77–21.77)
Hao et al. (2015)	China	Sino‐Tibetan	Mandarin	VC	898	13	19.67 (12–30)
Hung and Chang (2023)	Taiwan	Sino‐Tibetan	Mandarin	SP	117	3	27.33 (20–36)
Stoll et al. (2012)	Nepal	Tibeto‐Burman	Chintang	SP	53	53	35.98 (25–51)
Turkay (2005)	Turkey	Turkic	Turkish	SP	30	30	22.32 (17.1–27.87)
Avcu et al. (2014)	Turkey	Turkic	Turkish	SP	31	31	25.19 (13.5–36.8)
Kaygusuz and Zeyrek (2016)	Turkey	Turkic	Turkish	SP	2	2	21.5 (16–27)
Schults, Tulviste, and Konstabel (2012)	Estonia	Uralic	Estonian	VC	592	1	12 (12–12)
Schults and Tulviste (2015)	Estonia	Uralic	Estonian	VC	219	1	10.41 (10.41–10.41)
Olah (2004)	Hungary	Uralic	Hungarian	VC	89	1	20 (20–20)
Gentner (1982)	Multiple	Multiple	Multiple	SP, VC, Other	16	16	19.19 (14–29)
Au et al. (1994)	Multiple	Multiple	Multiple	VC	10	3	20.9 (19.25–23)
Tardif et al. (1997)	Multiple	Multiple	Multiple	SP	22	3	23.02 (21.77–24.35)
Tardif et al. (1999)	Multiple	Multiple	Multiple	SP, VC	143	6	17.6 (10.9–20.65)
M. Kim et al. (2000)	Multiple	Multiple	Multiple	VC	16	16	20.09 (16.11–24.9)
Levey and Cruz (2003)	US	Multiple	Multiple	VC	34	2	12 (12–12)
Bornstein et al. (2004)	Multiple	Multiple	Multiple	VC	269	7	20.29 (20.02–20.6)
Liu (2007)	Multiple	Multiple	Multiple	SP	72	12	36.76 (20.83–55)
Lee (2008)	Hong Kong	Multiple	Multiple	SP	4	4	17.75 (17–18.5)
Liu, Zhao, and Li (2008)	Multiple	Multiple	Multiple	SP	72	12	36.5 (18.5–54.5)
Lucas & Bernardo (2008)	Philippines	Multiple	Multiple	SP	120	2	40.2 (40.2–40.2)
Tardif et al. (2008)	Multiple	Multiple	Multiple	VC	967	3	11.45 (11.31–11.61)
Xuan (2010)	US	Multiple	Multiple	VC	100	2	26.9 (26.9–26.9)
Özcan, Altinkamiş, and Gillis (2016)	Belgium	Multiple	Multiple	VC	110	10	20.6 (9–33)
Sri Adnyani, Sutjiati Beratha, Pastika, and Suparwa (2017)	Indonesia	Multiple	Multiple	SP	6	6	26.33 (18–36)
Chai, Low, Wong, Onnis, and Mayor (2021)	Malaysia	Multiple	Multiple	VC	889	6	23.47 (22.5–24.8)
Setoh et al. (2021)	Malaysia	Multiple	Multiple	SP	60	2	19.3 (19.3–19.3)

*Note*. Country indicates the country of data collection, and language family and language indicates the language(s) studied (multicountry, multilanguage, and multilingual studies are marked as “multiple” as appropriate). Method values are as follows: VC = vocabulary checklist; SP = spontaneous production; Other = other method. *N* indicates the number of children in the original study and # Datapts. indicates the number of finest‐grained noun bias measurements we were able to extract from the paper (maximum of one per language per child per time sampled, e.g., for a longitudinal study of multilingual children). Age shows the mean age of the original study participants, along with the range (minimum–maximum); two studies lack age information.

**Fig. 4:**
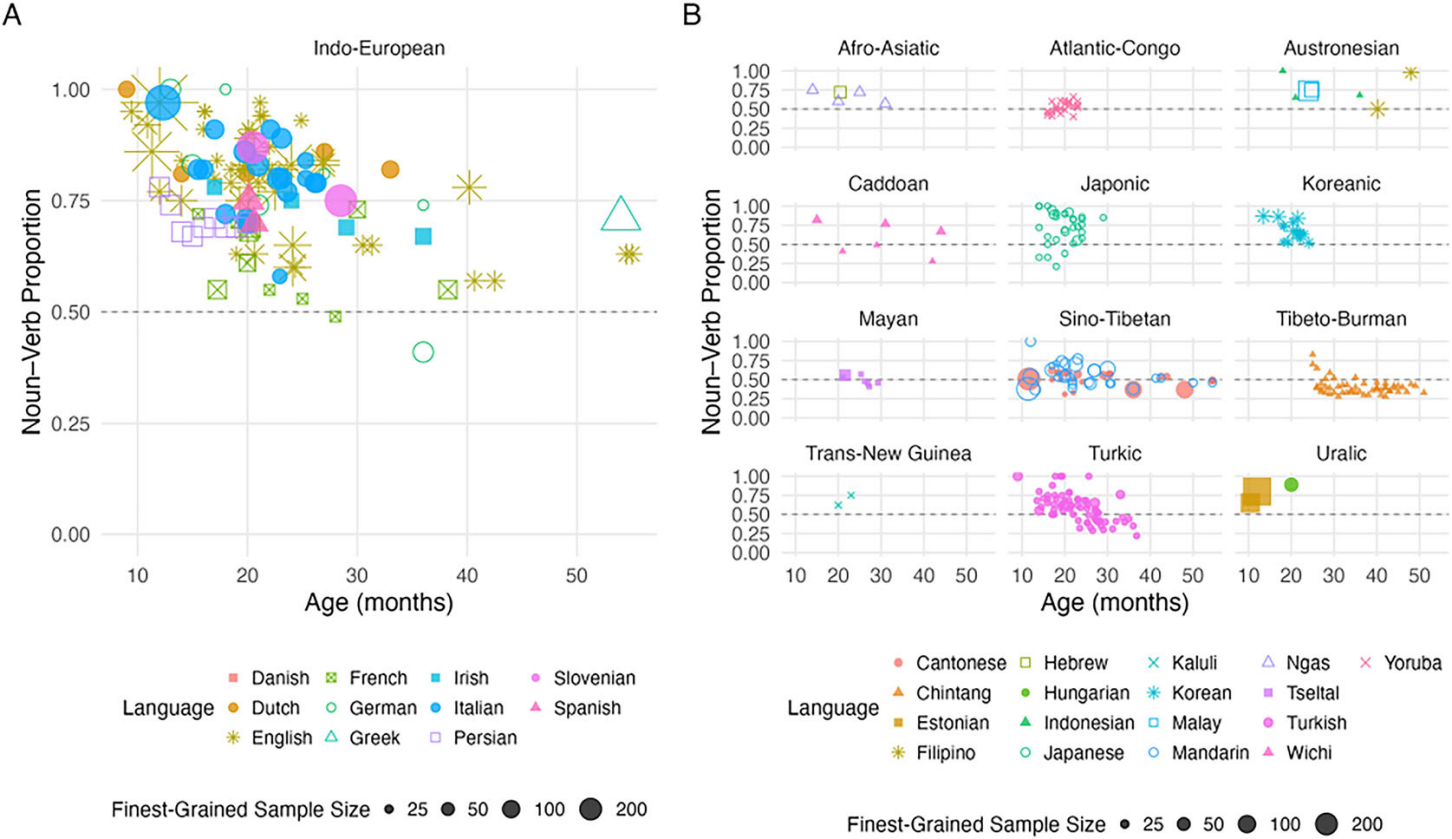
Distribution of noun‐verb proportion over age in sample languages. Panel (A) shows datapoints for Indo‐European languages, and Panel (B) shows datapoints for non‐Indo‐European languages, grouped by language family, in the sample. Each point represents an extracted datapoint of noun‐verb proportion at the given mean age in our sample. Point size reflects the finest‐grained sample size, or number of subjects represented, associated with each datapoint. Individual languages are represented by distinct combinations of point colors and shapes.

The average noun‐verb proportion across studies was 0.74 (range = 0–1; *SD* = 0.17), providing strong evidence for the noun bias, with moderate variability. Mean child age was 20.34 months (range = 9–55, *SD* = 9.88), around which time children begin to experience a vocabulary spurt and produce multiple new words daily (we anticipate a ∼90–308‐word vocabulary size at 18–24 months; Frank et al., 2021). Noun bias effects are hypothesized to be most apparent before reaching a 50‐word vocabulary inventory, which typically occurs before 16–20 months (Frank et al., 2021; Gentner, 1982; Gentner, 2006; Tardif et al., 2008; see also Table S2). While this hypothesis appears to hold broadly in our sample (Fig. 4, Panel A), not all languages show a clear age‐related decline in noun bias (Fig. 4, Panel B). We caution readers to *not* interpret these differences directly; many languages have few data points (or data only for limited age ranges), and the datasets are systematically skewed by language in the use of observational versus checklist methods, which alters noun bias estimates. Instead, mixed‐effects regression is more useful for understanding trends in the data while jointly accounting for this variability across datasets.

### 3.2 Mixed‐effects regression analysis

The mixed‐effects linear regression (lme4; D. Bates, Mächler, Bolker, & Walker, 2015) of noun‐verb proportion included fixed effects of noun morphology score, verb morphology score, BWO, fusional typology, age, publication year, bilingualism, research methodology, and random effects for individual papers and languages. The model also included two‐way interactions of noun morphology by age and verb morphology by age, to test whether these effects are weaker later in development, when children have better mastered early morphosyntactic representations (Table 2).

**Table 2:** Fixed effects from mixed effect regression analysis

	Estimate	Std. Err.	*t*‐value	*p*‐value
Intercept	−3.25	4.10	−0.79	.432
Publication year	0.00	0.00	0.66	.510
Bilingualism	0.01	0.04	0.31	.761
Age (centered)	0.00	0.00	0.59	.553
Basic Word Order: Verb‐Initial	−0.02	0.12	−0.19	.832
Basic Word Order: Verb‐Final	−0.12	0.07	−1.61	.114
Noun Morphology Score	0.46	0.17	2.60	.016^*^
Verb Morphology Score	0.04	0.16	0.22	.828
Fusional Morphology: No	0.03	0.08	0.38	.681
Method: Observation	−0.13	0.04	−3.66	< .001^***^
Method: Other	0.42	0.12	3.60	< .001^***^
Noun Morphology Score * Age (centered)	−0.01	0.00	−2.31	.022^*^
Verb Morphology Score * Age (centered)	−0.01	0.01	−3.00	.003^**^

* *p* < .05; ^**^ *p* < .01; ^***^ *p* < .001.

Larger noun bias was hypothesized to occur with: higher verb morphosyntax, lower noun morphosyntax, fusional morphology, verb‐medial BWO, younger age, and checklist‐based methods. Control variables (publication year and bilingualism) and random effects (language and paper) account for additional variability across studies. To reduce heteroscedasticity, noun‐verb proportions were log‐transformed in the model, which was also weighted by sample size.

Indeed, noun morphology significantly influenced the size of the noun bias, though in the opposite direction to what was hypothesized (β = 0.46, *SE* = 0.17, *t* = 2.60, *p* = .016). Verb morphology trended in a similar direction, but was not significant in the model (*p* = .828). As predicted, spontaneous production studies showed smaller noun biases compared to checklist studies (β = −0.13, *SE* = 0.04, *t* = −3.66, *p* < .001). Other research methods, including diary entries and interviews, showed larger noun biases compared to checklists (β = 0.42, *SE* = 0.12, *t* = 3.60, *p* < .001); however, the lack of coherence in this small collection of studies (three studies, <1% of participants) prevents any firm conclusions from this finding. Age showed a small negative interaction with both noun morphology (β = −0.01, *SE* = 0.00, *t* = −2.31, *p* = .022) and verb morphology (β = −0.01, *SE* = 0.01, *t* = −3.00, *p* = .003), suggesting a lesser influence of noun and verb morphology with age. No other significant effects were found.

### 3.3 Random forest analysis

An exploratory random forest approach was run with the suggested default of 500 trees using the R package randomForest (Liaw & Wiener, 2002). This analysis helps to identify which individual attributes are most closely associated with observed noun bias outcomes based on the data itself, distinct from a hypothesis‐driven approach. Random forest analyses use a machine learning algorithm to construct numerous decision trees and aggregate their outputs in making final predictions (Breiman, 2001). Each decision tree is built not only on a random subset of the original data (bootstrap aggregating, i.e., “bagging”) but also with a random subset of features (i.e., “feature bagging”), reducing risks of overfitting and increasing model robustness. Crucially, unlike traditional regression models, random forest approaches are nonparametric. While the feature importance output of such models inevitably suffers from diminished interpretability (e.g., missing any potential relationships between variables), these models are well‐suited for exploratory analyses. The present random forest analysis complements the theory‐driven regression above, incorporating all individual linguistic features extracted from Grambank as well as child age, method, and bilingualism.

**Fig. 5:**
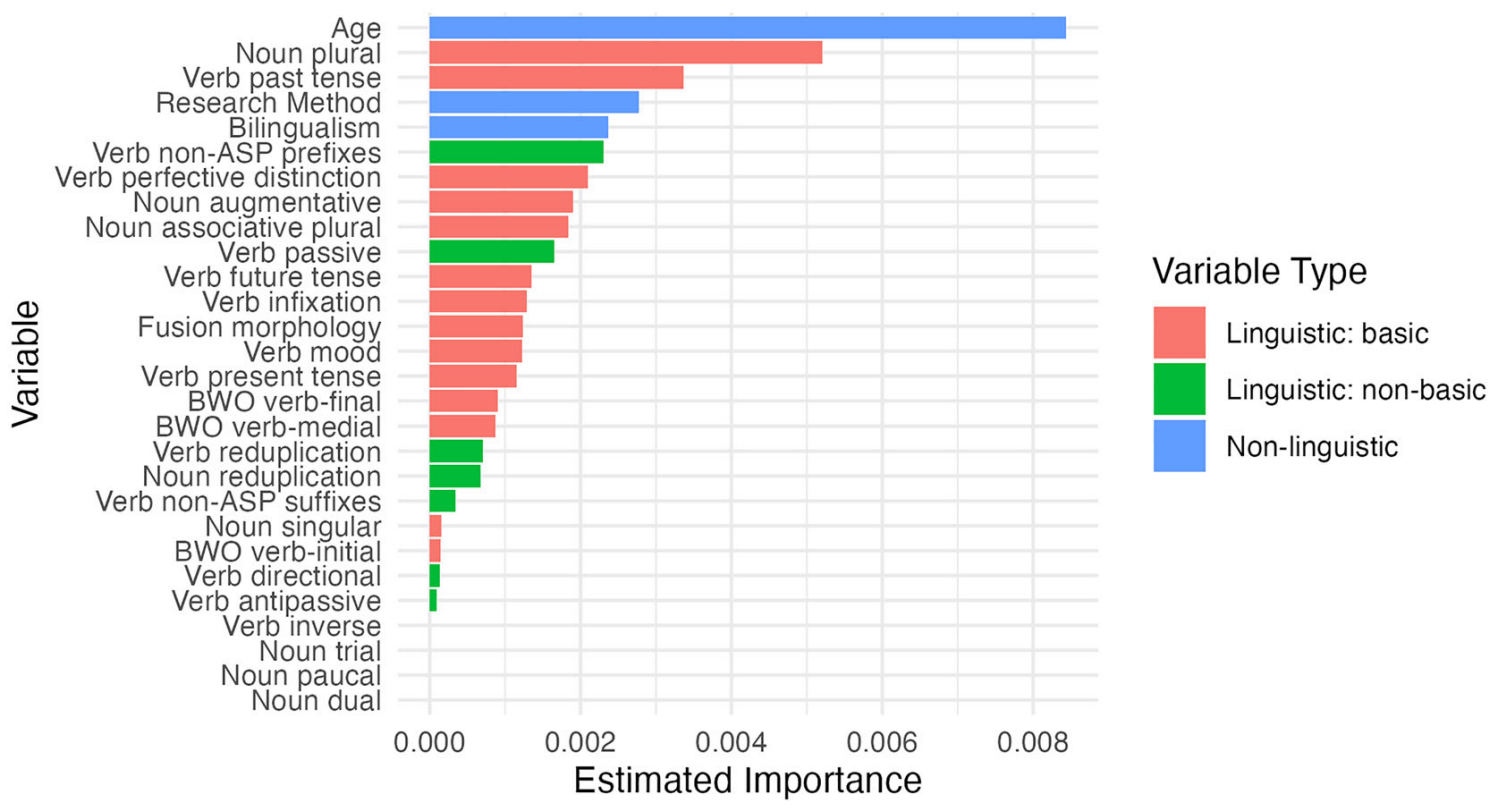
Estimated variable importance from random forest analysis. The “basic” linguistic features (pink) are those included in the Grambank‐derived noun and verb predictors used in the mixed‐effects regression analysis. The “nonbasic” linguistic features (green) are the *additional* morphosyntactic features for which we have Grambank data. The “nonlinguistic” features (blue) are a mixed group of demographic and methodological features.

The model had an *R* ^2^ (explained variance) of .57 and an MAE (deviation from out‐of‐bag predictions) of 0.10, suggesting a good fit. Fig. 5 shows estimated importance values, reflecting the amount of sample variation explained by each predictor. The top five important variables associated with greater noun bias were: being younger, the presence of plural marking on nouns (Grambank question GB044), the presence of past tense verb morphology (Grambank question GB083), use of checklist methods, and being monolingual.

## 4 Discussion

This paper synthesizes and quantitatively analyzes five decades of noun bias research. The findings generally confirm the existence of this bias, but also reveal variability in noun bias size as driven by linguistic and methodological factors. While the methodological effects were straightforward, the linguistic ones were not. For example, the regression outcomes confirmed a significant noun morphology effect, but in an unexpected direction: more noun morphology predicted *larger* observed noun bias. We discuss and speculate on each of these findings in turn, beginning with our findings regarding morphology.

### 4.1 Linguistic effects

Morphology could aid word learning in a variety of ways, including facilitating lexical stem segmentation and cueing a word's syntactic and semantic properties. From the perspective of stem segmentation, our findings could suggest that inflections serve as anchors that help identify noun stem boundaries; infants show evidence of using and recognizing inflectional morphology well before they begin to speak (Y. J. Kim & Sundara, 2021; Marquis & Shi, 2012; Mintz, 2013). In principle, similar benefits could extend to other syntactic categories if their inflectional patterns are regular and consistently available. However, if this anchoring process is critically augmented by the learner's parsing and interpretation of the inflectional morphology, object words may still maintain an advantage. Echoing the broader discussion by Gentner (1982; 2006) on objects’ greater perceptual availability, we note that the meanings carried by noun morphology may typically be more perceptually transparent than the meanings carried by verb morphology. For example, plurality marking often relates to readily observable, stable properties of entities (e.g., one vs. multiple objects), whereas TAM require the learner to track changing states over time and (invisible) causal relationships and actor perspectives. This pairing—the relatively greater perceptual availability of object referents *and* their morphology—may also jointly augment children's ability to use their emerging grammatical knowledge to constrain referent candidates and infer word meanings. At first glance, the random forest analysis might appear to support the speculation above—the Grambank feature for the presence of plural marking on nouns predicts a larger noun bias. However, this is the only nominal morphosyntactic feature that acts as a strong predictor of the noun bias (e.g., we see a low importance of augmentative marking, dual number marking, etc.). Further, the only other morphosyntactic feature with high “importance” pertains to verbs: past tense marking morphology is associated with a larger noun bias. Considering the lack of an effect of verb morphology in our mixed‐effects regression, and the lack of relevant further evidence in the random forest analysis, we are skeptical that verbal morphology plays much of a role in predicting noun bias size—at least in the present dataset. Notably, to the extent that either nominal or verbal morphology plays a role in the noun bias, our data point toward a reduction in their impact over the first few years of life.

BWO also does not predict the magnitude of the noun bias in our dataset, running counter to a possible “edge effect” advantage (Caselli et al., 1995; Gentner, 1982; 2006; Slobin, 1985). This absence of BWO effects was consistent across the regression and random forest analyses. The lack of BWO effects could be attributed to several factors. First, as a predictor of the noun bias, word order per se may be a red herring. Word order effects on the noun bias have primarily been discussed under the umbrella of “verb‐friendliness” (e.g., de León, 1999; Gentner, 1982; 2006; M. Kim et al., 2000; Tardif et al., 1997), where key languages with verb‐initial and verb‐final BWO (e.g., Tseltal and Korean) also maintain other verb‐friendly input features (e.g., baseline frequency: Sandhofer et al., 2000; morphosyntax: Gentner, 1982; Gleitman, 1990; social‐pragmatic factors: Tardif et al., 2008). It may be these other, co‐occurring verb‐friendly features (and not BWO) that produced lesser noun bias in prior work. Second, edge effects might prevail, but we would not see them here if our BWO values only roughly indicated of how often verbs and nouns meet the edge of an utterance. Word order is well understood to be variable, even within‐language, and analyzing languages by the gradience of their word order preference (rather than their predominant word order) has important implications for theoretical models of syntax, psycholinguistics, and language acquisition (Levshina et al., 2023). In short, relying on a language's “basic” word order—a categorical variable—may obscure crucial variation in the actual edge positioning of nouns and verbs in input across discourse contexts. Thus, the influence of pragmatic and processing‐related factors on argument elision and syntactic variation (e.g., single‐word utterances, question‐formatted utterances) is likely to provide a better account of the utterance‐edge frequencies of the nouns and verbs in children's input, across and within languages. Finally, our dataset may be underpowered to detect the effects of BWO. Verb‐medial order is noted as default (“unmarked”) in 16 of the sampled languages (57%), verb‐final in eight of the sampled languages (29%), and verb‐initial in only four of the sampled languages (14%; Filipino, Irish, Ngas, and Tseltal), with two languages noted in multiple categories (Spanish: verb‐medial and verb‐final; Irish: verb‐medial and verb‐initial). Thus, our fully non‐verb‐medial samples were rather small (verb‐final = 7; verb‐initial = 3), and so our ability to statistically detect impacts of BWO on noun bias magnitude was critically limited.

We draw two overall conclusions from this pattern of results, one more conservative and one more speculative. Our first, more conservative, conclusion is that linguistic factors only play a small role in predicting noun bias size. With no evidence for word order effects and only marginal evidence for morphosyntactic effects (limited to nouns, and diminishing over age), we lack a clear linguistic explanation for the apparent variability in noun bias observations across these 28 languages and 12 language families.

Consider, for example, the interaction effect of noun morphology and child age. If noun morphology is associated with greater noun bias because it is helpful for word learning, one would expect this effect to *increase* with age, given that children's sensitivity to morphology increases as they begin to learn about their language (Anglin, Miller, & Wakefield, 1993; Gleitman, 1990; Slobin, 1985). However, we see the opposite pattern: the interaction *decreases* with age, suggesting that morphology has its greatest impact on word learning early in development. By the same logic, we would expect to see greater verb morphology to be associated with a *verb* bias, but the data trend instead toward greater noun bias with greater verb morphology. It is, therefore, difficult to see how these effects of morphology might relate to sensitivity. What, then, should we make of the observed noun morphology effect? In line with our earlier comments, we suspect that this pattern might be attributed to the skewness of our sample—in this case, not only typologically, but culturally and economically. We observe a skew in our sample toward Western, higher‐income economies, which are also the communities in which sociocultural practices of object‐centered pedagogical practices in child‐directed speech may be especially common (e.g., Ochs & Kremer‐Sadlik, 2015; Schieffelin & Ochs, 1986). In our data, such societies are disproportionately clustered at the higher end of the noun morphology distribution, and, to a lesser extent, the verb morphology distribution. This introduces a potential confound between cultural‐economic factors and documented morphological characteristics in this dataset. That is, instead of noun morphology itself, the observed association may reflect broader sociocultural differences correlated with the languages represented in our sample. This issue highlights the limits of a systematic review that only maintains sparse metadata on each language community. A broader sampling of participant communities in future work would go a long way toward ameliorating this shortcoming.

Analyses of children's actual input would also surely bring us closer to understanding this variability (see, e.g., Braginsky et al., 2019; Caselli et al., 1995; Stoll et al., 2012; Tardif et al., 1997), Grambank provides a powerful intermediate tool for estimating the likelihood that children encounter these linguistic features in their input. Indeed, we sampled five languages from our dataset to check whether their Grambank‐derived morphosyntax proportions correlate with the morphosyntactic density of nouns and verbs in children's input. The five languages chosen—Danish, Dutch, Irish, Italian, and Mandarin—span our observed range of Grambank‐derived scores, and show a close correlation with the mean morphemic length of nouns and verbs in children's input (Noun morphology: *r* = .809; *p* = .097 and Verb morphology: *r* = .954; *p* = .012; see Fig. S2). Thus, we conclude that early in development, morphology has some, albeit minor, predictive value for noun bias size. Our second, more speculative, conclusion is that, when linguistic features *do* matter, their impact derives from the same conceptual bias thought to underlie the noun bias more generally: the greater perceptual accessibility of noun morphology (particularly plural marking) may yield benefits in noun stem segmentation, noun referent identification, and noun categorization.

As we have seen, there is a long line of research on the noun bias arguing that linguistic structure is likely to be influential in shaping children's early expressive vocabularies (e.g., Gentner, 1982; Stoll et al., 2012; Tardif et al., 1997, Tyler, 2004). Our present conclusions, based on a large, cross‐linguistic comparison, are inconsistent with this idea. However, our findings are far from the final word on the question of noun bias variation. Though we bring together data on the noun bias from the largest number of languages to date, our dataset still only includes a small and Indo‐European‐skewed sample of human languages. With broader sampling—or more intentional sampling, for example, Pye (2017)—we might be better positioned to comment on the full spectrum of variation in the linguistic features of interest: utterance‐edge appearance, word order, noun morphology, and verb morphology. And while our initial analyses suggest Grambank features are useful indicators of input morphology (Fig. S2), other linguistic features that have been proposed to matter for the bias (e.g., noun elision) are not easily measured with our present approach. Experimental work is also likely to play a key role in teasing apart these potential linguistic features, particularly considering the practical challenges of collecting and processing more linguistically diverse vocabulary checklists and natural observation data.

### 4.2 Methodological effects

Method matters when measuring the noun bias. Vindicating prior claims (Gentner, 2006; Pine, 1992; Pine et al., 1996; Tardif et al., 1999), we find that checklists really do tend to give larger noun bias results. Among our 131 checklist‐derived datapoints, only two (1.5%) show a noun‐verb proportion of 0.5 or less, both from Mandarin‐acquiring children (Tardif et al. (1999): *N* = 24; Age_mean_ = 13.1; NV_prop_ = 0.37; Tardif et al. (2008): *N* = 336; Age_mean_ = 11.43; NV_prop_ = 0.38). By comparison, 116 of the 269 (43%) observation‐based datapoints show a noun‐verb proportion of 0.5 or less. The nine languages in our dataset with both checklist *and* observational data show a uniformly positive advantage for checklists (mean noun‐verb proportion increases by 0.08–0.36, average = 0.16; in increasing order: Italian, Cantonese, German, Mandarin, Japanese, Turkish, English, French, Wichi). This pattern closely follows past work (e.g., Salerni et al., 2007; Tardif et al., 1999; Taverna & Waxman, 2020). Aggregating vast quantities of data from 27 checklists on 22 languages, Frank et al. (2021) also previously found that nouns are overrepresented in the early expressive vocabularies of children across the board: the *lowest* five relative representation scores go to three Mandarin checklists, one Cantonese checklist, and one Kiswahili checklist, all of them still above zero (i.e., showing noun overrepresentation). We thus conclude that method is the primary factor predicting noun bias magnitude. Why would method matter?

One explanation lies with caregivers’ reliability in reporting on their children's expressive vocabulary. Arunachalam and colleagues (2021) compare CDI responses by caregivers with U.S. English‐speaking children with and without autism spectrum disorder. Across both groups, they find more inconsistent caregiver responses for verbs than nouns, perhaps reflecting caregivers’ greater uncertainty in reporting children's verb knowledge. Such uncertainty might arise because caregivers themselves attend more to nouns during their interactions with children (Tardif et al., 1999), because the children's verbal productions are more variable, less mature, or less imageable than their nominal ones (Gillette et al., 1999; Gleitman et al., 2005), or because the checklist includes nouns that are somehow more salient to caregivers compared to the included verbs (Nelson et al., 1993; see also Arunachalam et al.’s (2021) discussion of “babiness”). If that is the case, future work might examine whether caregiver certainty for verbs is higher for caregivers who tend to focus more on actions during child‐centered interaction, as has been argued for Korean, Tseltal, and Tsotsil, among others (Casillas et al., 2024; Choi, 2000; de León, 1999).

Moreover, observational measures are more subjective to context effects than cumulative parental reports because they capture only a selection of children's total vocabulary production. The context in which the speech is sampled can impact what types of words are pragmatically invited, which shapes the appearance of a noun bias (Pine et al., 1996; Tardif et al., 1999). For instance, book‐reading contexts tend to encourage object labeling, which would lead to more noun production, whereas play contexts are relatively more likely to highlight actions, therefore, eliciting more verbs (Tardif et al., 1999). Speech sampled in observational studies of word production may be overrepresented by more verb‐eliciting contexts, such as naturalistic play sessions, therefore, yielding higher relative verb proportions than more cumulative activity data would show. In turn, parental reports might be more context‐weighted, as parents aggregate vocabulary use across situations. Nevertheless, it is worth noting the possibility that parental report instruments like the CDI may sample nouns more exhaustively than the other word types, potentially leading to a sampling overrepresentation of nouns in the checklist relative to children's underlying vocabularies.

Another, more speculative explanation relates to the differential likelihood that frequent verbs and nouns are used across the day, rather than in context‐specific settings. To explain this idea, we will step through a thought experiment. Let us assume that a given observational session typically captures a single activity (e.g., a bout of caregiver−child toy play). We also assume that, for short activity recordings in a given community, we can calculate a fairly stable ratio of unique verb types to unique noun types (e.g., a “typical” session might capture children's production of four unique verbs and seven unique nouns; noun‐verb proportion = 0.63). If we sampled a second activity during the day, we would expect to get a similar noun‐verb proportion (0.63), but would be likely to observe some new noun and verb stems—stems we did not see in the first activity that grow our cumulatively observed noun and verb inventories. The key question is, then, how much overlap there is in the verbs used across activities versus the nouns. If we assume that frequently used nouns are more context‐specific than frequently used verbs are, we would expect to see more overlap in the verbs used across activities compared to the nouns used across activities. In this example, let us imagine that we observe 1–2 new verb stems and 2–6 new noun stems for every new activity. Thus, the observed noun inventory will grow faster than the observed verb inventory and, concomitantly, our noun‐verb proportion will climb higher the more data we observe. If we simulate this scenario over 100 contexts, we climb from an initial noun‐verb proportion of 0.63 to nearly 0.73 (see Fig. S3). Turning back to vocabulary checklists, retrospective caregiver reports are valuable tools precisely *because* responders are drawing on immense and cumulative experience with their child. If we consider the caregiver's internal catalogue of child word uses to approximate something like our many‐activities simulation, we would expect the noun‐verb proportion to be higher than what we would observe from any single activity, even when caregivers are perfect reporters. Of course, this hypothetical outcome depends on the differential context‐specificity of common verbs versus nouns, which may be marginal and (we imagine) susceptible to cross‐community variability. For the time being, we only wish to demonstrate that some of the sampling assumptions inherent in traditional observational and checklist methods may help to explain the systematically higher noun bias estimates found in the latter.

### 4.3 Limitations

There are some important limitations to the current systematic review. First, we included age as a continuous predictor in our analyses. Our aim was to capture broad developmental trends in the noun bias while also preserving coverage of the relevant literature. We chose age rather than total vocabulary size because it is the most consistently reported developmental variable across studies in the dataset; 97% of eligible studies reported child age in some form, while only 66% reported a total vocabulary size. That said, chronological age is a limited proxy of development—children of the same age may differ substantially in vocabulary size and composition, and other developmental indices may better serve to illuminate typical learning trajectories. Additionally, we treat age as a linear variable, but the influence of age and the mechanisms shaping the noun bias across age may change as children's vocabularies grow. Across development (in age or total vocabulary), we note that experimental work will play a critical role in examining the moment‐by‐moment mechanisms by which children glean information about new words from linguistic signals.

Second, the granularity of extracted data points necessarily varied across studies. Although we coded data to the finest level of detail available, some studies only reported group‐level averages rather than individual data points. In these cases, the noun‐verb proportions computed were associated with an average age and total sample size rather than an individual's age and sample size, obscuring some within‐sample variability. While our modeling approaches account for this heterogeneity by the weighting of sample sizes and the inclusion of random effects, this limitation on granularity cannot be overcome. This issue reflects a constraint in the existing literature and highlights the importance of more consistent reporting of individual‐level data (e.g., via anonymized data on repositories hosted by the Center for Open Science, https://www.cos.io/).

Additionally, our systematic review relied on specific, theory‐driven search terms intended to identify work that directly engaged with the noun bias debate. Our assumption was that most work with any findings relevant to the debate—either aligning or conflicting with the noun bias—would mention the noun bias debate at some point. Indeed, among the 406 noun‐verb proportion data points extracted, 111 (27%) exhibited a verb bias (e.g., Stoll et al., 2012; Miyata et al., 2003; Avcu et al., 2014). Our present approach used search terms specific to the noun bias, and is thus narrow for the sake of prioritizing study relevance. Our search terms did *not* guarantee comprehensive coverage of all relevant studies; a study that contained relevant extractable data but did not mention the noun bias is likely to have been excluded. Indeed, we conducted an alternative, broader search to ensure our search was not overly narrow (see Footnote 1)—our review of 600 papers turned up one missed reference that would have been included had we found it with our narrower search (Gruendel, 1977). And we are aware that others have been missed (e.g., Papaeliou & Rescorla, 2011). However, we can think of no clear reason to expect that the excluded studies would differ systematically from the empirical patterns of those included (Table 1). Taken together, the current search strategy represents a deliberate balance between literature coverage and topical specificity. Our findings should, therefore, be interpreted as reflecting patterns within the retrieved literature, rather than providing an exhaustive account of all research on early word learning biases. Further, the present findings are, by design, primarily limited to checklist and observational approaches, and are thus ultimately best complemented by experiments and work examining communication and shared attention in real‐world interactions.

## 5 Conclusion

The present study systematically reviewed 68 papers spanning 28 languages, 12 language families, and 5 decades of research on the noun bias, using two statistical approaches: mixed‐effects regression and a random forest analysis. The regression showed that both greater noun morphology and checklist methods predict larger noun bias outcomes. The random forest analysis identified (younger) age, the presence of nominal plural marking, the presence of verbal past‐tense marking, monolingualism, and checklist use as the five most important predictors of larger noun bias. We conclude that methodological factors far exceed linguistic ones in influencing the magnitude of observed noun bias and, tentatively, that linguistic effects may be limited to noun morphology in early development. The findings in this review are, however, critically limited by an urban and Indo‐European skew in the language communities sampled, best remedied by future research with multimethod approaches that target broader and more intentional sampling of developmental contexts. Such future work will be key to understanding how and when interactional practices and linguistic features influence children's propensity to learn “hard” words.

## Supporting information



Supporting Information

## Notes

1 These proportions for basic word order, which come from Grambank v2, differ somewhat from those reported in Hammarström (2016). For individual languages with BWO data, they originally report slightly lower proportions of verb‐medial and verb‐initial languages, with a higher proportion of verb‐final ones: 41.1% (verb‐medial); 12.9% (verb‐initial); 43.7% (verb‐final); 2.4% (no dominant order). Because some language families are over‐represented, their reported proportions by language *family* differ substantially, showing dramatically lower representation for verb‐medial orders and higher representation for verb‐final ones: 15.8% (verb‐medial); 11.5% (verb‐initial); 65.6% (verb‐final); 7.1% (no dominant order).

2 To verify that our search was not overly narrow, we conducted a second search using broader terms (i.e., “word types” OR “early words” OR “early vocabulary” OR “expressive vocabulary”), which yielded an immense literature (>34k references). We screened stratified random samples (*n* = 600) of references across the same decades studied in the present work (1970s–2020s) using the same eligibility criteria. The majority of retrieved studies under broader search terms were not relevant (*n* = 548, 91.3%). Among relevant references, *all but one* would have been excluded from analysis for: no extractable data (*n* = 18), text not written in English (*n* = 15), using a conceptual/computational approach (*n* = 6), featuring a nontypically developing population (*n* = 5), focusing on experimental data (*n* = 3), or other (*n* = 4).

3 Our primary dataset spanned infancy through 5 years of age. Although the noun bias is often discussed as most relevant to children's earliest expressive vocabularies (e.g., Gentner, 1982, 2006; Tardif et al., 2008), our initial literature search suggested substantial variability in the age ranges at which the noun bias was assessed, and large‐scale data from Wordbank (Frank, Braginsky, Yurovsky, & Marchman, 2021, Chapter 11) suggest that the noun bias may persist through at least the end of toddlerhood (∼30 months). For additional focus on the earliest developmental stage, we report a supplementary analysis restricted to our youngest participants (≤ 14 months; Table S2).

4 Grambank gave us six out of six basic verb morphology features for 25 of the languages (89%) and five out of six for the remaining three languages; as well as six out of six basic noun morphology features for 23 of the languages (82%), five out of six for two languages, four out of six for one language, and three out of six for two languages.
