# Supplementary material for: Cross‐Cultural Variation in the Noun Bias in Early Vocabulary Development: A Systematic Review
Source: Cogn Sci. 2026 Jul 9;50(7):e70241. doi: 10.1111/cogs.70241 (PMC13347766; doi:10.1111/cogs.70241)
Supplement: Supplementary file 1 — Supporting Information [file COGS-50-e70241-s001.docx]

Cross-cultural Variation in the Noun Bias in Early Vocabulary Development: A Systematic Review (Supporting Information)

Yiqun Zhang^1,2^, Subin Kim^2^, & Marisa Casillas^2^

^1^ University of Pennsylvania

^2^ University of Chicago

Correspondence concerning this article should be addressed to Yiqun Zhang, Dept. of Psychology, 425 S. University Ave., Philadelphia, PA 19104-6018, United States. E-mail: [miazh@sas.upenn.edu](mailto:miazh@sas.upenn.edu)

**Supporting Information**

SI Fig. 1.

*Decision Tree (rpart) Complementing the Random Forest Analysis. To visualize the hierarchical interactions and directional effects of the predictors identified in the Random Forest (RF) analysis, a separate, deterministic decision tree was generated using recursive partitioning (“rpart” package; Milborrow, 2026). While the RF model provides an aggregate measure of global variable importance across 500 trees, this single decision tree provides a representative snapshot of how those variables interact. Each internal node represents a binary split on the predictor. For the terminal nodes, the top values are the predicted noun-verb proportions for that node and the bottom percentages are the proportion of observations associated with that node. Node color reflects the magnitude of predicted noun biases, with warmer colors indicating higher noun biases and cooler colors indicating lower noun bias; N = noun and V = verbs. Consistent with the RF results, the tree relies on age, methodological, and morphological markers to partition the data. Note that where variables are highly redundant (e.g., Verb past tense and Verb mood), the decision tree algorithm selects the single most efficient predictor for visualization. Observations were weighted by sample size to account for differences in study-level sampling.*


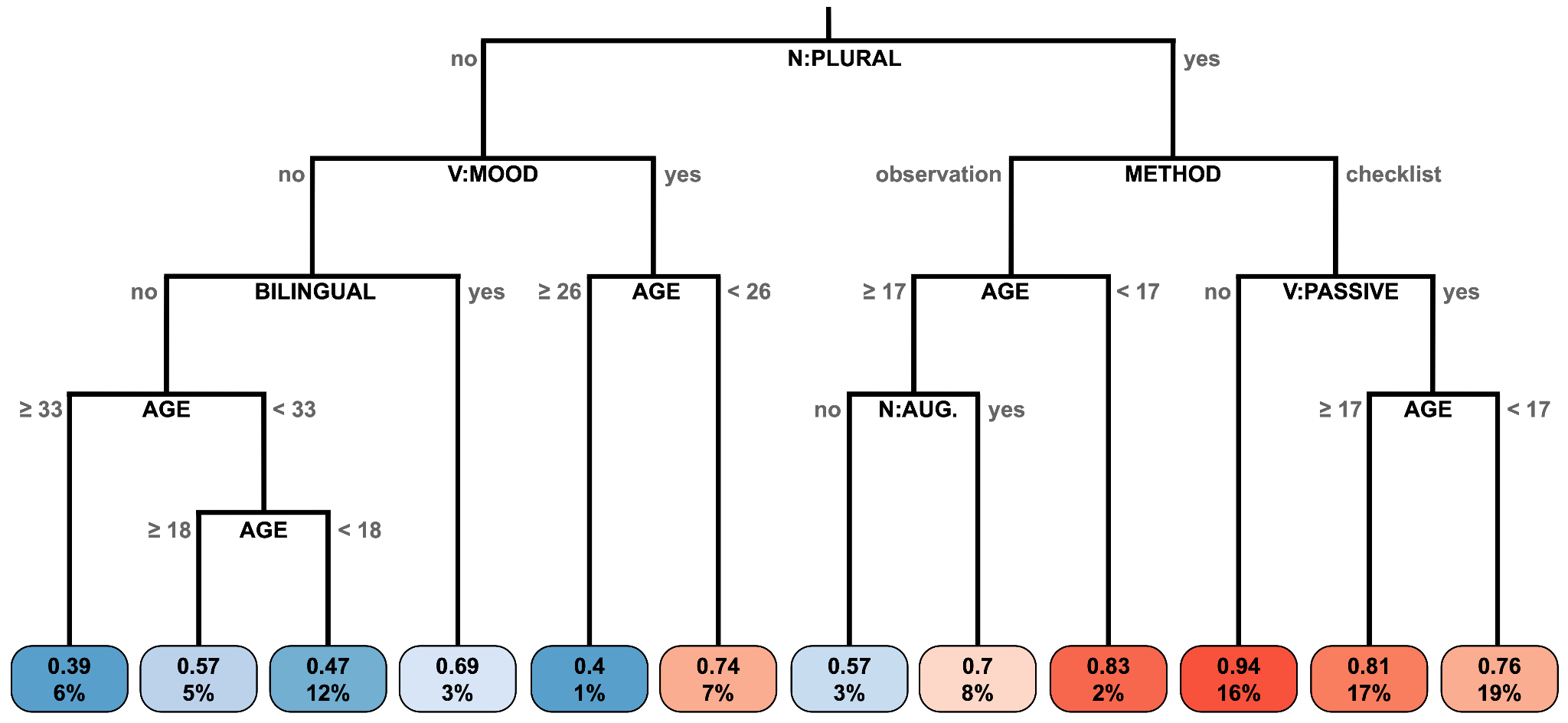


SI Table 1.

*The table displays the 25 morphosyntactic features relevant to the current study that were extracted from Grambank (*[*https://grambank.clld.org/*](https://grambank.clld.org/)*; Skirgård et al., 2023). The features were categorized into three Types: noun morphology (N), verb morphology (V), and basic word order (BWO). Features related to basic word order (BWO), nominal number marking, noun diminutization/augmentation, and basic tense, aspect, and mood (TAM) expression were included in the main mixed-effect regression analysis (Regression = ‘yes’). In contrast, all of the listed features (basic and not) were included in the random forest analysis.*

| **ID** | **Question** | **Type** | **Regression** |
| --- | --- | --- | --- |
| GB042 | Is there productive overt morphological singular marking on nouns? | N | yes |
| GB043 | Is there productive morphological dual marking on nouns? | N | yes |
| GB044 | Is there productive morphological plural marking on nouns? | N | yes |
| GB046 | Is there an associative plural marker for nouns? | N | yes |
| GB159 | Are nouns reduplicated? | N | no |
| GB165 | Is there productive morphological trial marking on nouns? | N | no |
| GB166 | Is there productive morphological paucal marking on nouns? | N | no |
| GB187 | Is there any productive diminutive marking on the noun (exclude marking by system of nominal classification only)? | N | yes |
| GB188 | Is there any productive augmentative marking on the noun (exclude marking by system of nominal classification only)? | N | yes |
| GB158 | Are verbs reduplicated? | V | no |
| GB079 | Do verbs have prefixes/proclitics, other than those that only mark A, S or P (do include portmanteau: A & S + TAM)? | V | no |
| GB080 | Do verbs have suffixes/enclitics, other than those that only mark A, S or P (do include portmanteau: A & S + TAM)? | V | no |
| GB081 | Is there productive infixation in verbs? | V | yes |
| GB082 | Is there overt morphological marking of present tense on verbs? | V | yes |
| GB083 | Is there overt morphological marking on the verb dedicated to past tense? | V | yes |
| GB084 | Is there overt morphological marking on the verb dedicated to future tense? | V | yes |
| GB086 | Is a morphological distinction between perfective and imperfective aspect available on verbs? | V | yes |
| GB108 | Is there directional or locative morphological marking on verbs? | V | no |
| GB147 | Is there a morphological passive marked on the lexical verb? | V | no |
| GB148 | Is there a morphological antipassive marked on the lexical verb? | V | no |
| GB149 | Is there a morphologically marked inverse on verbs? | V | no |
| GB312 | Is there overt morphological marking on the verb dedicated to mood? | V | yes |
| GB131 | Is a pragmatically unmarked constituent order verb-initial for transitive clauses? | BWO | no |
| GB132 | Is a pragmatically unmarked constituent order verb-medial for transitive clauses? | BWO | no |
| GB133 | Is a pragmatically unmarked constituent order verb-final for transitive clauses? | BWO | no |

SI Table 2.

*Fixed Effects from Mixed Effect Regression Analysis: Youngest Sample*

*This model was fit to a subset of the data including only data points with a mean age of 14 months or younger, corresponding to an approximate productive vocabulary size of 10 words (estimated based on Wordbank data retrieved mid-December 2025). The model included all predictors from the main analysis, excluding the main effect of age and its interactions with noun and verb morphology. This supplementary model is based on N = 31 extracted datapoints from 17 papers and 12 languages.*

| **Predictor** | **Estimate** | **Std. Err.** | ***t*-value** | ***p*-value** |
| --- | --- | --- | --- | --- |
| Intercept | -1.97 | 15.68 | -0.13 | .902 |
| Publication Year | 0.00 | 0.01 | 0.11 | .918 |
| Bilingualism | 0.22 | 0.19 | 1.15 | .266 |
| Basic Word Order: Verb-Initial | 0.18 | 0.54 | 0.34 | .742 |
| Basic Word Order: Verb-Final | -0.05 | 0.30 | -0.17 | .872 |
| Noun Morphology Score | 0.16 | 0.88 | 0.18 | .864 |
| Verb Morphology Score | 0.36 | 0.54 | 0.66 | .553 |
| Fusion Morphology: No | -0.27 | 0.37 | -0.74 | .502 |
| Method: Observational | -0.07 | 0.19 | -0.37 | .718 |
| Method: Other | -0.10 | 1.05 | -0.10 | .923 |

*Note.* ⁎p<0.05; ⁎⁎p<0.01; ⁎⁎⁎p<0.001

SI Table 3.

*Fixed Effects from Mixed Effect Regression Analysis with the Addition of Pro-drop Feature*

*This model was fit to the same data used for the main analysis. The model included all predictors from the main analysis, with the addition of the pro-drop feature derived from Grambank (Feature 522: Can the S or A argument be omitted from a pragmatically unmarked clause when the referent is inferrable from context ("pro-drop" or "null anaphora”)) as a predictor.*

| **Predictor** | **Estimate** | **Std. Err.** | ***t*-value** | ***p*-value** |
| --- | --- | --- | --- | --- |
| Intercept | -2.59 | 4.22 | -0.61 | .541 |
| Publication Year | 0.00 | 0.00 | 0.49 | .627 |
| Bilingualism | 0.02 | 0.04 | 0.46 | .648 |
| Age (centered) | 0.00 | 0.00 | 0.17 | .869 |
| Pro-Drop: Yes | -0.13 | 0.08 | -1.63 | .129 |
| Basic Word Order: Verb-Initial | -0.04 | 0.13 | -0.28 | .782 |
| Basic Word Order: Verb-Final | -0.10 | 0.09 | -1.13 | .267 |
| Noun Morphology Score | 0.50 | 0.20 | 2.50 | .029* |
| Verb Morphology Score | 0.12 | 0.17 | 0.68 | .509 |
| Fusion Morphology: No | 0.05 | 0.09 | 0.63 | .534 |
| Noun Morphology Score * Age (centered) | -0.01 | 0.01 | -1.44 | .153 |
| Verb Morphology Score * Age (centered) | -0.01 | 0.01 | -2.48 | .014* |
| Method: Observational | -0.13 | 0.04 | -3.42 | < .001*** |
| Method: Other | 0.42 | 0.13 | 3.34 | .001** |

*Note.* ⁎p<0.05; ⁎⁎p<0.01; ⁎⁎⁎p<0.001

SI Fig. 2.

*Grambank noun/verb scores plotted against CHILDES mean morphemes per word measure. Five languages from our dataset that also have transcript data on CHILDES (Danish, Dutch, Irish, Italian, and Mandarin) were selected to represent the spectrum of morphological variability observed in Grambank noun and verb scores. For each language, a maximum of 10 transcripts were sampled from each family across all available naturalistic CHILDES corpora (MacWhinney, 2000). Analysis was restricted to non-target-child speakers. For transcripts lacking morphological data, the Batchalign pipeline (Liu et al., 2023) was used to generate morphology tiers.*

*
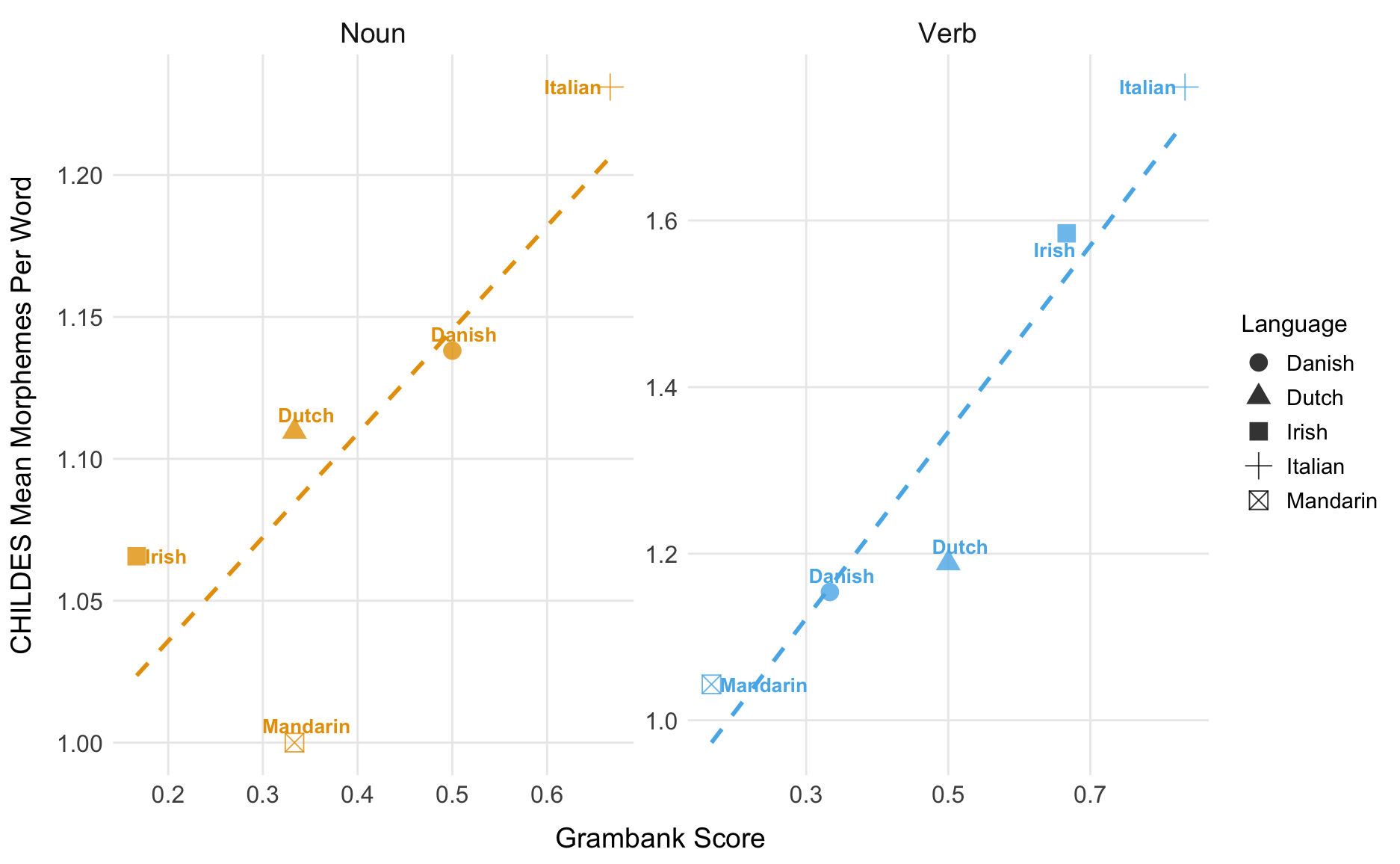
*

SI Fig. 3.

*Hypothetical increase in observed noun-verb proportion as we observe more activity contexts. In this toy simulation, any individual activity context will give us a noun-verb proportion of 0.63 (4 verbs and 7 nouns), but with each additional context we accumulate new noun and verb stems. We simulate the situation in which frequent verbs are used across a wide variety of activities while frequent nouns are used in a more context-specific way: we observe 1–2 new verbs per new activity and 2–6 new nouns per activity. Across 100 activity contexts, we cumulatively see more new nouns than verbs (e.g., totaling 416 nouns and 154 verbs; a noun-verb proportion of 0.73). If we run this simulation 50 times, we see the same pattern: a stable within-activity noun-verb proportion of 0.63 but cumulative data suggesting a substantially larger noun-verb proportion: a mean of 0.726 across runs (SD = 0.013).*

*
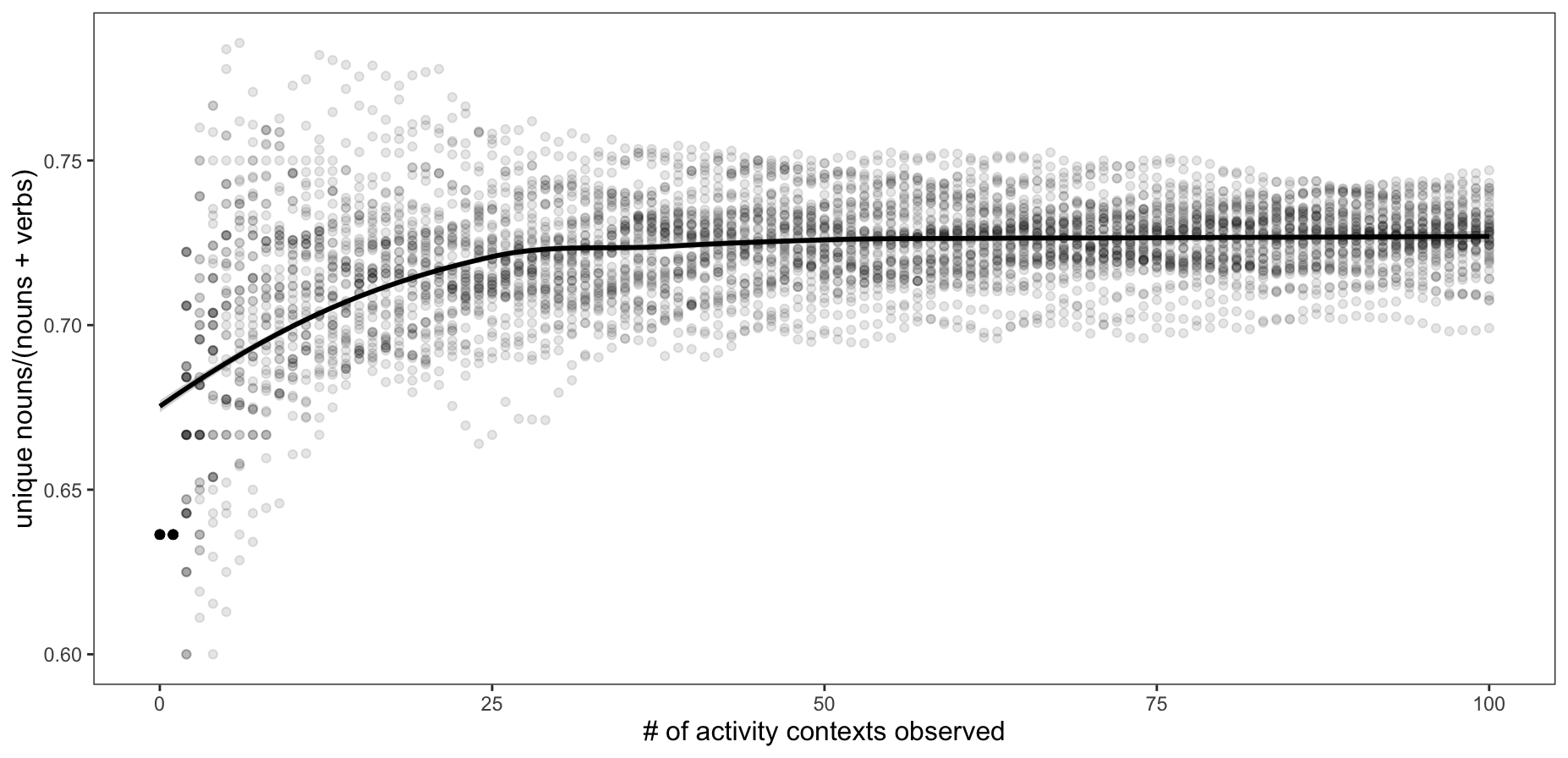
*

**References**

Liu, H., MacWhinney, B., Fromm, D., & Lanzi, A. (2023). Automation of language sample analysis. *Journal of Speech, Language, and Hearing Research*, *66*(7), 2421–2433. <https://doi.org/10.1044/2023_JSLHR-22-0064>

MacWhinney, B. (2000). *The CHILDES Project: Tools for analyzing talk* (3^rd^ Edition). Mahwah, NJ: Lawrence Erlbaum Associates.

Milborrow S. (2026). *rpart.plot: Plot 'rpart' Models: An Enhanced Version of 'plot.rpart'.* R package version 3.1.4, [https://CRAN.R-project.org/package=rpart.plot](https://cran.r-project.org/package=rpart.plot)

Skirgård, H., Haynie, H. J., Hammarström, H., Blasi, D. E., Collins, J., Latarche, J., Lesage, J., Weber, T., Witzlack-Makarevich, A., Dunn, M., Reesink, G., Singer, R., Bowern, C., Epps, P., Hill, J., Vesakoski, O., Abbas, N. K., Ananth, S., Auer, D., … Gray, R. D. (2023). *Grambank v1.0.* Zenodo. https://doi.org/10.5281/ZENODO.7740140
